# Poly (1-butene-*ran*-ethylene) Monomodal Copolymers from Metallocene Catalysts: Structural and Morphological Differences with Increasing Ethylene Content

**DOI:** 10.3390/polym11071133

**Published:** 2019-07-03

**Authors:** Carla Marega, Federica Malizia, Stefano Spataro

**Affiliations:** 1 Department of Chemical Sciences, via Marzolo 1, University of Padova, 35131 Padova, Italy; 2 Basell Poliolefine Italia Srl, P.le G. Donegani 12, 44100 Ferrara, Italy

**Keywords:** 1-butene/ethylene copolymers, polymorphism, thermal analysis, X-ray diffraction, microscopy, hot melt adhesive

## Abstract

Samples of random poly(butene-*ran*-ethylene) copolymers produced with metallocene catalysts were studied in order to elucidate the different behaviors of this particular class of materials as a function of increasing ethylene (C2) content. The samples cooled down from the melt are semi-crystalline or amorphous and crystallize in different crystal modifications, depending on the amount of C2. Thermal analysis, X-ray diffraction, and microscopic techniques were used to follow the changes of the materials with aging time and to understand the structural and morphological behavior with the aim of highlighting possible peculiar properties, which may be of great interest in the application of such materials in the field of Hot Melt adhesives.

## 1. Introduction

The first synthesis of isotactic Poly(1-butene) (PB) was reported in 1954 by Natta and collaborators [1], starting from heterogeneous solid catalysts (Ziegler–Natta catalysts) already used for the production of isotactic polypropylene, and they obtained a linear semi-crystalline polymer with high stereoregularity from the polymerization of 1-butene.

The polymerization of 1-butene can be also carried out using metallocene catalytic systems [2], and the use of such a catalyst ensures a better random and uniform distribution of ethylene with respect to that achieved with Ziegler–Natta catalysts. Therefore, the choice of the catalytic approach, in the synthesis of PB, is an important step in the design of the polymer.

Poly(1-butene) exhibits a singular and very complex polymorphic behavior: according to the conditions under which the crystallization process is conducted, five crystalline forms can be obtained [3,4] which differ in terms of unit cell, helical conformation [5,6] (Table 1), thermodynamic stability and the consequent chemical, physical, and mechanical properties.

Form I is the most thermodynamically stable species at room temperature. Although form II is the most favored species from the kinetic point of view, it is a metastable form which is obtained by cooling the melted polymer and tends to transform spontaneously into form I.

Form I’ can be obtained by crystallization from the melt under high pressure conditions [7], in the presence of suitable solvents [4] or by low temperature polymerization [8]. This form is not distinguishable from form I by wide angle X-ray diffraction, as they share the same crystalline cell, but they differ by the melting temperature (Tm) (form I’ presents a lower Tm) [9] and they exhibit different characteristic peak intensities in IR spectra [4].

Form II’ is obtained by crystallization from the melt at a pressure above 2000 atm [10]. Also, in this case, the diffraction pattern is the same as in form II, while Tm is lower.

Form I’ and II’ are called ’imperfect’ forms, respectively, of form I and form II.

Form III, orthorhombic, can be obtained by precipitation at room temperature, from a solution of the polymer in organic solvents [11,12,13,14,15,16,17]. It is generally considered a metastable form which, and in the presence of low heating rates, tends to crystallize in form II and in form I’ [14]. An increase of temperature promotes the transition III→II [13].

One of the ways to speed up the solid-to-solid transformation II→I is to copolymerize 1-butene with short-chain α-olefins [18,19,20]. In particular, the presence of ethylene accelerates such transition until the direct crystallization from the melt of the stable form I for the most modified copolymers. 

It was observed that, as a function of the amount of comonomer and the experimental conditions (temperature, pressure, cooling rate) in which the molten polymer is cooled, the rate of transformation is not affected by different molecular weight, but the presence of ethylene comonomer enhances the rate of the transformation II→I. In particular, the most modified and defective the copolymer molecules are, the lower the time needed for the interconversion at *T*_room_ [20,21]. Using metallocene catalysts that allow to control the content and the type of chain defects (stereo and regio defects), it has been possible to clarify the relationship between the microstructure of the chain and the polymorphic behavior of both PB and 1-butene/ethylene copolymers [22,23,24]. On one hand, the presence of ethylene promotes the melt-crystallization of form I’, on the other hand, it accelerates the transformation of form II into form I. For low amounts of comonomer (around 1%), the molten polymer crystallizes in form II, which over time turns into Form I at room temperature.

Copolymers with an intermediate ethylene content (around 2% wt) crystallize in both forms I’ and II and transform afterwards to form I. On the other hand, more modified copolymers (with C2 > 3% wt) do not crystallize from the melt, but they crystallize from amorphous state upon aging directly in form I and I’. In particular, for the most modified copolymers, a peculiar characteristic was found, that is the ability to be undergo "self-sealing" after being cut. This effect has been attributed to the high mobility of the polymer chains due to the low melting temperature of form I’ and to the low degree of crystallinity of the copolymer [25].

The presence of ethylene, in addition to acting on the polymorphic behavior of the PB, involves some variations in the properties of the polymer such as the decrease of the crystallization rate, the degree of crystallinity and the melting temperature. Its influence on the mechanical properties of the copolymer is also noteworthy: the increase in the comonomer content follows the decrease in the glass transition temperature, the hardness and the breaking load while increasing flexibility and elasticity [23,25].

The focus of this paper is to characterize poly (1-butene-*ran*-ethylene) copolymers, with different ethylene contents, which are the building blocks of a more complex system that is generated by using a setup of two-reactor in series. 

This material has applications as a Hot Melt Adhesive (HMA). Within this application, there is a complex system of end uses that are mainly driven by viscosity and crystallinity, where the polymer is the major part of a blend formed also by a tackifier and a polymeric wax. Today the application is covered by amorphous poly(alpha-olefin)s (APAOs), poly (ethylene-vinyl acetate) (EVA) and propylene and ethylene (C2)-based plastomers [26,27]. The role of a polyolefin in a HMA formula is the core that gives strength and tackiness. It should have adhesive and cohesive properties, so a combination of two polymers is the ideal system. 

The analysis has involved several techniques that allowed a structural and morphological characterization of the different samples, the first was achieved by wide angle X-ray diffraction (WAXD) related to thermal analysis (DTA), while the second one was, instead, performed by small angle X-ray diffraction (SAXS) and transmission electron microscopy (TEM).

## 2. Materials and Methods

The samples taken into consideration were obtained from pilot plants in LyondellBasell G. Natta Research Centre in Ferrara (Italy) by using Zr-based metallocene catalysis [2] and they consisted of 1-butene modified with different ethylene (C2) content, as described in Table 2, which gathers the main characteristics of the obtained materials. The polymerization was carried out in liquid monomer of 1-butene at 70 °C in a single continuous reactor to produce random copolymers with a weight percentage of ethylene going between 0 and 20% (Scheme S1 in Supporting Information).

The use of PB is growing in applications that require softness and tenacity. With the development of new catalysts, like the Zr-based metallocene, we can obtain polymers with lower molecular weight with respect to the standard Ziegler–Natta products and better copolymerize the comonomer content (ethylene in this case), so that real plastomers having no crystallinity could be successfully obtained.

The materials have an increasing C2 content (0, 1, 2.4, 2.9, 3.8, 6.8% by weight, see Figure S1) and almost constant viscosity (MFI). This allows us to study the effect of the different compositions while fixing the viscosity parameter. As expected, density and *T*g are directly linked to C2 content randomized in the chain, while average molecular weight and MFI are fixed. The low polydispersity index is a direct consequence of the catalyst used to polymerize these samples.

### 2.1. Thermal Analysis

The calorimetric measurements were carried out by using roughly 6 mg of polymers enclosed into aluminum crucibles by means of TA Instrument Q2000 instrument operating under nitrogen atmosphere. The temperature scale and enthalpy were calibrated by using high purity indium as a standard. 

The main thermal protocol involved a first heating of the samples at 10 °C/min from room temperature to 180 °C and an isothermal step at this temperature for 5 minutes (in order to erase completely their physical and mechanical history) followed by a cooling step at 10 °C/min down to −20 °C. The DSC crucible was then kept at room temperature and atmospheric pressure for different times ready to be melted up to 180 °C at 10 °C/min after different aging times.

### 2.2. Wide Angle X-ray Diffraction

WAXD patterns were recorded in the diffraction angular range 5–35 °2θ by using a Bruker D8 Advance Powder Diffractometer (Germany), working in the reflection geometry, and CuKα radiation was used.

WAXD analysis was carried out on the films of 1 mm thickness, prepared in DTA and conditioned at room temperature and atmospheric pressure for different aging time.

The application of the least-squares fit procedure elaborated by Hindeleh and Johnson [28] gave the degree of crystallinity by weight (CWAXD), calculated for the samples at the end of the solid–solid transformation.

### 2.3. Small Angle X-ray Scattering

The SAXS measurements were performed in a MBraun system by using CuKα radiation from a Philips PW1830 X-ray generator (The Netherlands). The patterns were recorded by a position sensitive detector in the scattering angular range 0.1–5.0 °2θ and corrected for the blank scattering. A constant, continuous background scattering [29] was subtracted and the obtained intensity values $\tilde{I}$ (*s*) were smoothed, in the tail region, with the aid of the *s*$\tilde{I}$ (*s*) versus 1/*s*^2^ plot [30]. Then Vonk’s desmearing procedure [31] was applied and the one-dimensional scattering function was obtained using the Lorentz correction: I_1_(s) = 4πs^2^I(s), where I_1_(s) is the one-dimensional scattering function and I(s) is the desmeared intensity function.

The sum of the average thicknesses of the crystalline and amorphous layers was determined as the Bragg identity period L of the function I_1_(s).

#### SAXS Data Analysis

The evaluation of the SAXS patterns according to some theoretical distribution models [32] was carried out referring to the Hosemann model [33], which assumes the presence of lamellar stacks having an infinite side dimension. This assumption takes into account a monodimensional electron density change along the normal direction to the lamellae.

The fitting procedure [32] of the calculated one dimensional scattering function with the experimental one allows one to optimize the values of the thicknesses and distributions of the crystalline and amorphous layers, the long period and the crystallinity, along with their distribution, associated with lamellar stacks.

### 2.4. Transmission Electron Microscopy 

TEM images were obtained using a FEI Tecnai 10 (USA), operating at an acceleration voltage of 100 kV and with a resolution of 0.34 nm.

Using a cryogenic microtome (Leica EM UC7, Germany), a thin layer (100 nm) was sectioned from the aged sample at −70 °C. This instrument operates at a temperature lower than the glass transition temperature of the sample, thus allowing the morphology of the sample to be maintained and improving the cutting conditions.

## 3. Results and Discussion

### 3.1. Structural Analysis: DTA and WAXD Measurements

In this study, the results obtained from wide angle X-ray diffraction were compared to those from thermal analysis, as the crystallization procedure applied to the samples were the same for specimens subjected to WAXD as well as DTA analysis.

**Figure 1:**
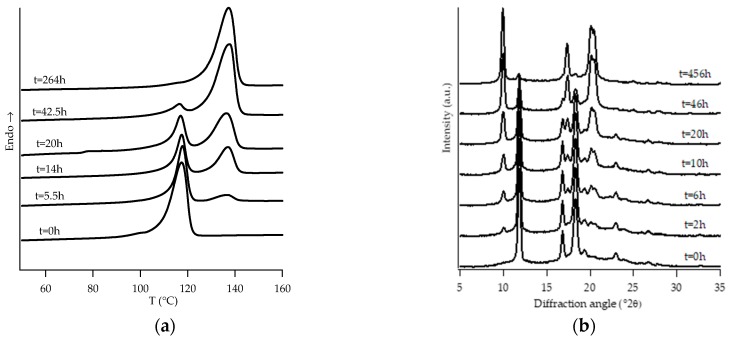
DTA thermograms (**a**) and WAXD patterns (**b**) of PB0 at different aging times.

**Figure 2:**
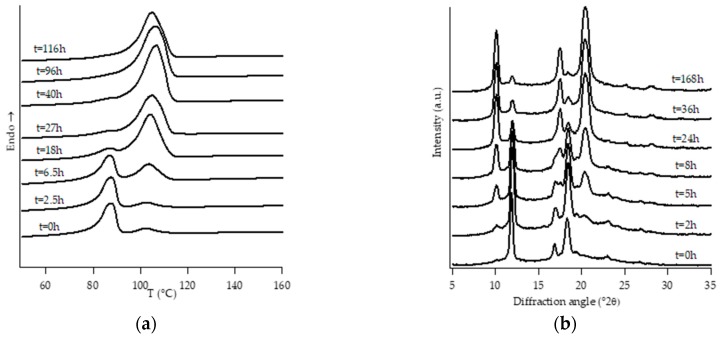
DTA thermograms (**a**) and WAXD patterns (**b**) of PB1 at different aging times.

**Figure 3:**
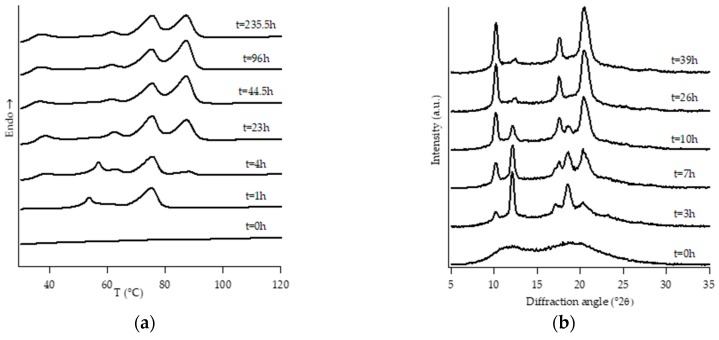
DTA thermograms (**a**) and WAXD patterns (**b**) of PB2.4 at different aging times.

**Figure 4:**
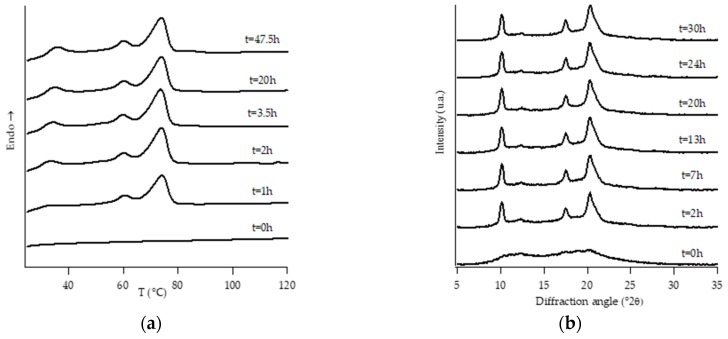
DTA thermograms (**a**) and WAXD patterns (**b**) of PB2.9 at different aging times.

**Figure 5:**
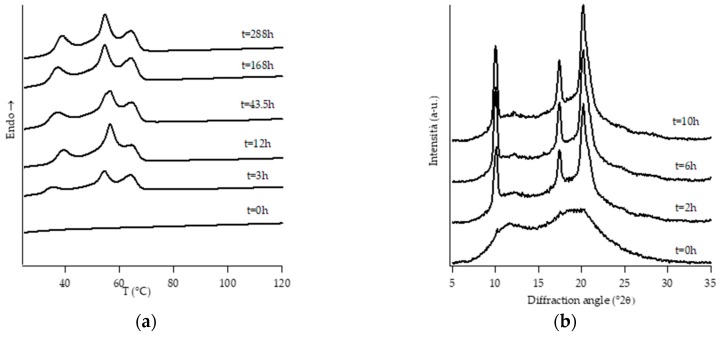
DTA thermograms (**a**) and WAXD patterns (**b**) of PB3.8 at different aging times.

**Figure 6:**
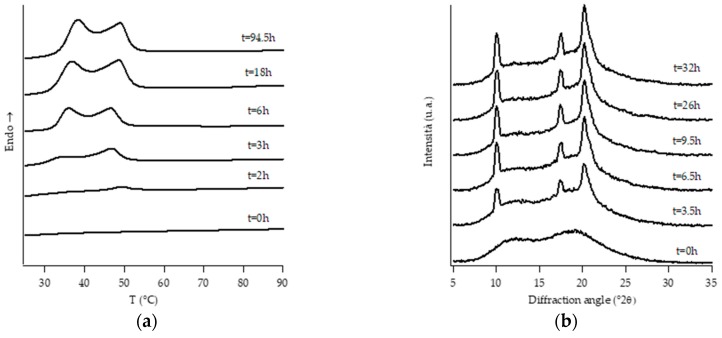
DTA thermograms (**a**) and WAXD patterns (**b**) of PB6.8 at different aging times.

Figure 1, Figure 2, Figure 3, Figure 4, Figure 5 and Figure 6 show, for every considered material, the collection of DTA heating scans (a), and WAXD patterns (b) recorded after having aged the specimens for different times at room temperature and atmospheric pressure.

Looking at DTA thermograms, except for samples PB0 and PB1, where just two endothermic peaks are visible, the other samples exhibit quite complex behavior, showing multiple endothermic peaks, which appear and change in intensity as a function of the aging time of the samples.

The ascription of such thermal peaks to the various polymorphic forms of PB was carried out by merging the information obtained from the analysis of the evolution of WAXD patterns of the corresponding sample, as well as from the literature data [23].

The homopolymer sample and the one with the lowest amount of C2 (i.e., PB0 and PB1, Figure 1 and Figure 2, respectively) show very similar evolution of crystalline phases during annealing, after their crystallization from the molten state. Both such samples crystallize from the melt mainly into form II, which then turns in form I as the ageing time increases.

On the other hand, all the other samples were completely amorphous after the controlled solidification from the melt and their successive structural evolution depended on the amount of C2. In particular, sample PB2.4 shows the typical development of form I that starts after the decreasing of form II, while the samples with the higher amount of C2 (i.e., PB2.9, PB3.8 and PB6.8) undergo to a spontaneous solid-phase transformation directly in form I.

However, it must be noted that, while the evolution of diffraction patterns is quite simple to understand, the thermal profiles exhibit multiple endothermic peaks whose nature is not straightforward and immediately associated with the various polymorphic phases of PB.

In the case of sample PB2.4, looking at the collection of DTA and WAXD data (Figure 3), the endotherms present at temperatures below 60 °C can be attributed to form II without any reasonable doubt due to the fact that they disappear as the annealing time increases. The other endotherms present at higher temperatures would, instead, be associated with form I, as diffraction data clearly show. However, in order to understand if the presence of multiple endotherms is due to the phenomenon of melting and crystallization of metastable crystals, followed by the crystallization of a more perfect one, or the melting of population of crystals with different lamellar thickness, DTA melting runs, collected at different heating rates on completely aged samples, have been executed (Figure S1). Since the heating rate has no influence on the relative temperature position of endothermic peaks as well as on the relative melting enthalpies, such endotherms cannot be attributed to the relaxation process of metastable crystals, but to the presence of crystals of form I with different lamellar thicknesses [34]. On the other hand, the small endothermic peak around 40 °C which appears after some annealing time is ascribed to form I’. In fact, this form is not distinguishable from form I in the diffraction pattern because these two forms present the same crystalline cell, but it is known [3,4] that they differ in the melting temperature. In particular, form I’ melts at a lower temperature with respect to form I, because it is characterized by a less perfect three-dimensional order [23].

Diffraction patterns of samples PB2.9, PB3.8 and PB6.8 show just the presence of diffraction peaks which are typical of crystallographic planes of form I. However, also for such samples, the thermal profiles are much more complicated, exhibiting several endothermic peaks. The assignation of these peaks has been carried out studying the thermal profiles of the completely aged samples obtained changing the heating rate.

The endothermic peaks of samples PB2.9 and PB3.8 (Figure S2), tend to get closer as the heating rate increases, meaning that the phenomenon of molecular reorganization occurs during the thermal scanning. In contrast, the endothermic peaks of sample PB6.8 (Figure S3) do not change both their relative temperature positions and enthalpies. This implies that the two endotherms at about 40 °C and 50 °C are related to the presence of form I’ and form I, respectively.

Table 3 summarizes the melting peak temperatures, assigned to the different polymorphic forms, together with the associated enthalpies and the degree of crystallinity calculated both by DTA and WAXD analysis, for all the considered materials.

Both from the results obtained by WAXD and thermal analysis, it can be observed that the increase in the ethylene content causes a decrease in the degree of crystallinity. In addition, a higher amount of C2 in the copolymer speeds up the phase transition: the sample PB2.4 transforms into form I faster than the others [35]. It is also possible to observe that the ΔHI and the TmI are influenced by the percentage of C2 present in the sample, in particular, as the C2 content increases, both parameters decrease. The same thing also occurs for the melting temperature related to form II, in the samples in which it is detectable. As for the Tm of form I’, it is not affected by the amount of C2, it remains in fact stable even at rather high C2 content.

The presence of ethylene produces an important modification in the structure of the sample. In particular, the increase of ethylene amount causes a significant decrease of the melting temperatures of form I and form II as well as of the degree of crystallinity; at the same time, it favors the kinetic of the spontaneous transformation of form II in form I. On the other hand, samples with C2 content equal or greater than 2.9%wt do not crystallize by cooling the melt to room temperature, but the amorphous samples crystallize directly in stable form I and I’ by aging at room temperature.

**Figure 7:**
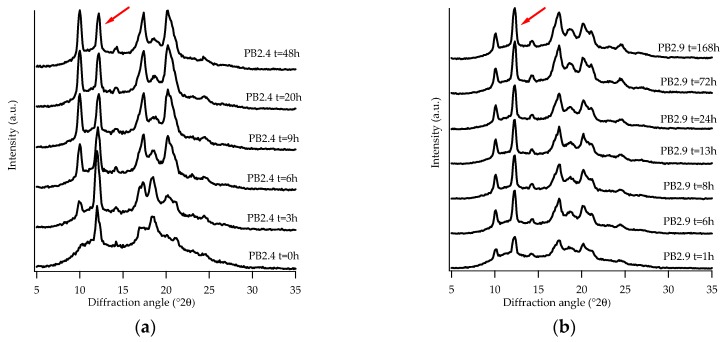
WAXD patterns of sample PB2.4 (**a**) and PB2.9 (**b**) subjected to pressure. (Red arrows indicate the (101)III peak after completion of phase transformation).

An unexpected behavior was noted for the samples PB2.4 and PB2.9, which under specific conditions showed the presence of form III. Although not strictly related to the purpose of the study reported in this paper, we found interesting the experimental observations concerning the presence of a form that has always been detected only starting from PB solution [11,12]. This occurred by applying a pressure of about 0.006 kg/cm^2^ on the samples immediately after the thermal treatment and prior to the WAXD analysis. Figure 7a,b show the diffraction profiles collected.

The presence of the crystalline form III is quite difficult to identify because its most intense peak, at about 2θ = 12.2 °, corresponding to the family of planes (101) III [36], overlaps with that of form II (2θ = 11.9 °). Nevertheless, what has allowed its identification in the diffraction profiles was the strange behavior of this peak with the increasing of aging time; in fact, an increase in intensity was noticed as the aging time increased, until a constant intensity was reached. This behavior could not be attributed to form II, which exhibits a decrease in intensity with increasing aging time. Further confirmation of the presence of the form III is given by the existence of a peak at about 2θ = 14.2 ° [36], which is typical of this crystalline form.

As can be seen from Figure 7a in this case, unlike what can be seen in the same sample not subjected to pressure (Figure 3b), the peak at 11.9 ° is already visible at *t* = 0, its intensity increases, at first, with increasing aging time and after about six hours it decreases and finally remains constant. This behavior is due to the overlap of the peaks of form II and III: at the beginning form II is the predominant one, as aging proceeds, it is converted into form I and, therefore, only form III contributes to the intensity of the peak.

Therefore, from the diffraction patterns it is possible to see that, at the end of the solid-phase transformation, there is a coexistence of form I and form III.

Sample PB2.9 (Figure 7b), due to the higher amount of C2, does not show the peak of form II. In this case, form I and form III are formed immediately from the amorphous and remain stable over time.

What has been found is that form III is not only obtainable from a PB solution in organic solvents [11,12], but also from the melt by pressure of the newly solidified sample. However, this occurs only for copolymer, as the examined ones, having intermediate amounts of C2, a not very high molecular weight, and a narrow molecular weight distribution (Table 2). These latter aspects could cause a greater mobility of the chains which, in combination with a favorable ethylene content, would lead to the formation of phase III, remaining stable over time at room temperature. 

### 3.2. Morphological Analysis: SAXS and TEM

The morphological investigation was carried out using two different techniques: small angle X-ray scattering and transmission electron microscopy. Both techniques, by exploiting different phenomena, provide information related to the morphology, and thus, it is possible to compare the data obtained via these methods. Using SAXS, the information related to the lamellar stacks is determined by elaborating the diffraction data using theoretical models. In contrast, the analysis of the TEM images can provide information regarding the morphology of a sample.

**Figure 8:**
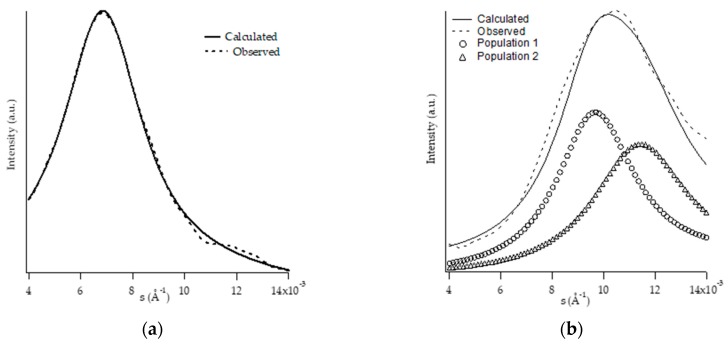
SAXS pattern (dotted line) and trace calculated by fitting procedure (solid line) of PB1 (**a**) and PB2.4 (**b**).

SAXS patterns of all samples were collected at the end of the aging process. Figure 8a,b shows, for the samples PB1 and PB2.4, the experimental and calculated diffraction patterns, the latter of which were obtained by reproducing the experimental data by means of theoretical models. The choice of the model leads to the reconstruction of the experimental profile through the optimization of the morphological parameters.

The profiles calculated for PB0 and PB1 samples (Figure 8a) were obtained by applying a model considering stacks of infinite dimension, and a Gaussian function was used for the distribution of the amorphous and crystalline areas. In both cases the best results are obtained using only one lamellar population.

Regarding the calculated profiles of PB2.4 (Figure 8b), PB2.9, PB3.8, and PB6.8 samples, the same model has been applied, but it was necessary to use two different lamellar populations to obtain a good fitting.

All the morphological data obtained by SAXS analysis are reported in Table 4.

**Figure 9:**
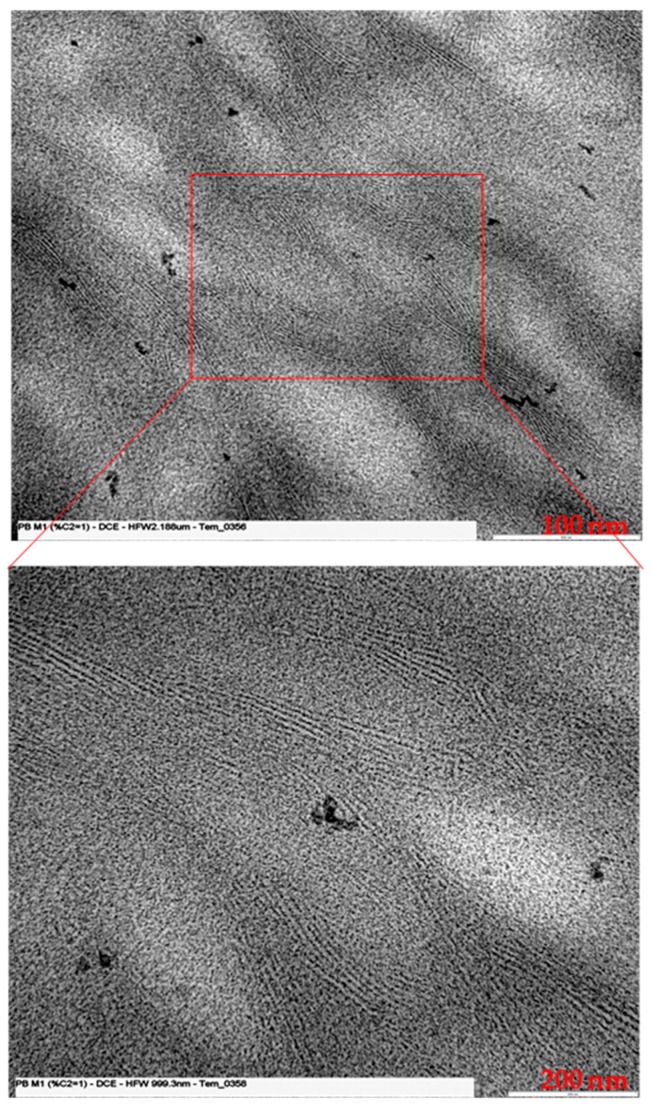
TEM image of PB1 at different magnification.

TEM images were also acquired for the samples at the end of the aging process. The measurement of the lamellar thicknesses, in order to obtain an average value more representative of the whole material, was carried out in different areas of the image, and on different images for each sample. Figure 9 shows the TEM image obtained for the PB1 sample, at two different magnifications, in which the lamellar morphology is clearly visible.

**Figure 10:**
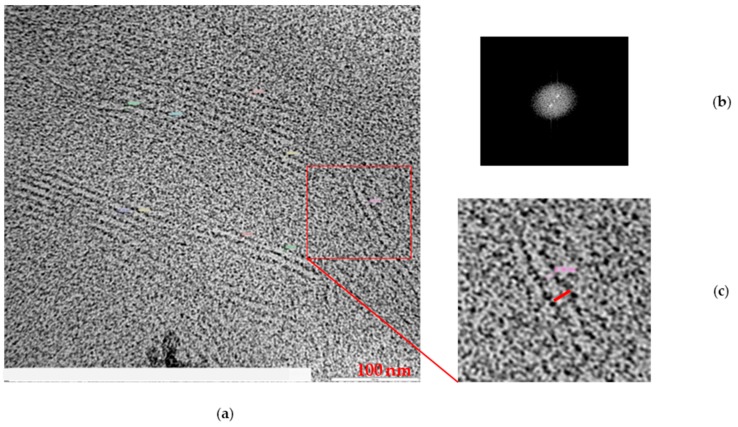
TEM image of PB1 (**a**), FFT signal (**b**), lamella image (**c**).

The measurement of the long period was obtained from the Fourier transform (FFT) of the signal: after selecting an area within the acquired image, with a processing program associated with the instrument, the FFT provided information regarding the variation of the signal as a function of the frequency (see sample PB1, Figure 10). If there is some periodicity in the morphology of the material, two spikes were observed in the FFT of the signal (white dots in Figure 10), which denote the existence of areas in which the signal repeats with a higher frequency. The distance between the center and the spike provides the average long period measurement.

**Figure 11:**
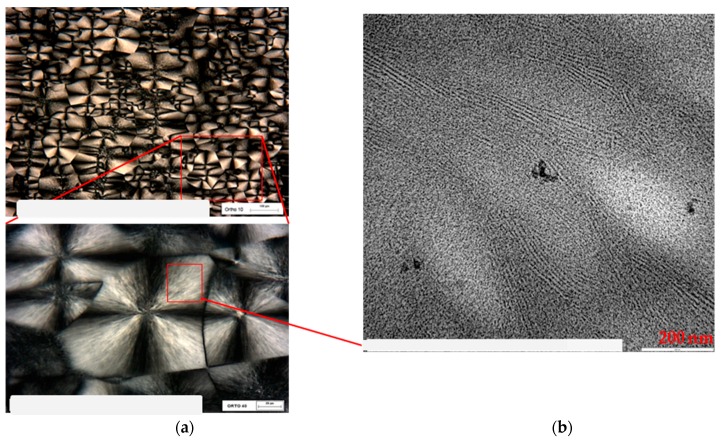
POM image of PB1 (**a**) at different magnification, and TEM image of the selected area (**b**).

Together with TEM analysis, the optical microscopy observation in polarized light was carried out on PB1, which is the copolymer presenting the higher degree of order. This further study allows us to explain more clearly the correlation between spherulitic and lamellar morphology. Figure 11a shows the images obtained through POM (Polarized Optical Microscopy), at two different magnifications, where the spherulitic morphology of the PB1 sample is clearly visible. Observing Figure 11b, collected by TEM in the region of the spherulite indicated in the red box, it is possible to highlight the lamellar morphology inside the spherulite.

Analyzing the data shown in Table 5, which collects the results obtained by SAXS and TEM, it is possible to draw interesting considerations. 

The parameters determined by SAXS, show a good agreement with those obtained by the microscopic analysis. Regarding PB0 and PB1 samples, the long period values obtained by the two techniques are very similar, also the lamellar thickness values obtained by SAXS are included in the interval determined by TEM analysis.

More difficult is the comparison of the other samples, which are characterized by the presence of two lamellar populations in the SAXS patterns. This morphology, in fact, was not revealed by TEM images. Using microscopy, it is possible to visualize the lamellar stacks, but not to carry out a statistical analysis on large areas of the sample that could lead to the identification of two distinct families of lamellar stacks containing lamellae and amorphous layers with average thicknesses such as to determine different long periods. Instead, the determination of the dimensions of the crystalline lamellae as well as of the long period has to be done by calculating an average value on all the stacks present without the possibility of discriminating those belonging to one or the other family. This then leads to a value of long period that is the average on all the stacks present in the images. Meanwhile, by applying SAXS, which allows analysis of a macroscopic area of the material, it is possible to discriminate the two lamellar populations and the two different values of long period characteristic of each one.

In Table 5, it can be seen that, for samples PB2.9, PB3.8, and PB6.8, the long period calculated by TEM, is similar to that determined by SAXS for one of the two populations and that the lamellar thickness is generally overestimated. This is probably due to the fact that the elaboration of the signal of TEM images is limited to very small areas of the sample and the contrast between dark and light areas is not enough to allow highly accurate measurements of the lamellar thicknesses.

Starting from the knowledge of the crystalline forms present in the samples, the two identified lamellar populations were attributed to forms I and I’; the samples presenting the double population are indeed those in which both phase I and phase I’ were observed. These crystalline forms, although presenting the same cell have, possess a different three-dimensional organization: form I’ has a low degree of order [23]. This characteristic probably has a great influence on the order at a morphological level as well, and so, form I and I’ are distinguishable by SAXS but not by WAXD. The population presenting the values of crystalline thickness and of higher periodicity (and therefore more similar to the homopolymer) was attributed to the form I, the other as a consequence to the form I’. From the integration of the areas of the calculated patterns, corresponding to the two populations, it was also possible to determine which of the two was more present in the samples. In all three samples, the prevailing population is the one with the highest lamellar thickness and periodicity, and therefore it can be concluded that, in all these samples, form I prevails.

## 4. Conclusions

The goal of this paper was the structural and morphological study of poly (1-butene-*ran*-ethylene) copolymers with different amount of ethylene, a characteristic which, as it is well known, influences the crystallization behavior and, consequently, the final properties of materials. 

Our interest for such samples lies in the fact that they are the building blocks of biphasic materials currently produced by using two reactors in series for hot melt adhesive application. In fact, the two different phases should deliver their individual properties to the whole final material. Therefore, the knowledge of their characteristics and behaviors should help in designing materials with the desired combination of adhesive and cohesive strength.

The structural study (WAXD and DTA) of the samples provided insight regarding the characteristic behaviors as a function of the ethylene content: for the homopolymer (PB0) and the sample with low C2 content (PB1) it was possible to observe the presence of the crystalline form II already in the polymer solidified from the melt and at short aging times and, with increasing time, the transformation into form I. The PB2.4 sample solidifies in an amorphous form and, with time, turns into form II and I, the latter increases over time while form II decreases. It has been confirmed that the increase of C2 accelerates the transformation II→I but decreases the crystallization rate. The samples with higher C2 content (PB2.9, PB3.8, and PB6.8) crystallize, starting from the amorphous solid, directly in form I. All samples, starting from PB2.4, present form I’, which can be distinguished from form I, due to the lower melting temperature. 

Thus, the C2 content clearly modifies the crystallization process, a propensity to solidify in the amorphous phase is noted and the crystallization takes place after a certain time of aging. Moreover, if the C2 amount is sufficiently high the material crystallizes directly in form I and I’.

It was confirmed that the increase of C2 accelerates the transformation, but decreases the crystallization rate.

A peculiar behavior was detected for the samples PB2.4 and PB2.9: by applying a modest pressure on the newly solidified material, form III was produced. It is possible to hypothesize that for random copolymers having such a molecular weight and distribution, an amount of ethylene between 2.3 and 3.0% by weight can lead to the generation of the form III starting from the melt.

By morphological analysis (SAXS and TEM), it was possible to determine the parameters relating to the lamellar stacks in the different samples. This investigation showed that the increase in the C2 content causes a noticeable decrease in the size of the crystalline lamella starting from the sample containing 2.4% of C2, which also results in a decrease in the long period, but which increases in the most modified sample in ethylene (PB6.8) and therefore shows a periodicity very similar to that of the homopolymer. This is a consequence of the increase in the thickness of the amorphous areas, as is evident considering the degree of crystallinity Φ_SAXS_, which decreases as the percentage of comonomer increases.

Of particular interest is the identification of a lamellar population characteristic of the form I’ and distinguishable from that of the form I, which allows us to establish that, although the two forms share the same crystalline structure, they can be distinguished by the different lamellar morphology as well as for the melting temperature. In particular, the presence of form I’ can be considered essential for industrial products, like HMA, whose applications benefit from the presence of a phase with a low melting temperature.

## Figures and Tables

**Table 1:** Crystallographic data [6] and references therein.

	Crystal Lattice	Helix	a (nm)	b (nm)	c (nm)
I/I’	Hexagonal	3/1	1.77	1.77	0.65
II/II’	Tetragonal	11/3	1.46	1.46	2.12
III	Orthorhombic	4/1	1.25	0.89	0.76

**Table 2:** Samples characterization.

		PB0	PB1	PB2.4	PB2.9	PB3.8	PB6.8
C2	%wt	0	0.8	2.4	2.9	3.8	6.8
MFI	g/10’	40–60	40–60	40–60	40–60	40–60	40–60
${\bar{M}}_{w}$ *		126151	126724	143923	131228	135570	141250
${\bar{M}}_{n}$ *		61755	62100	66768	61506	59634	65949
${\bar{M}}_{w}/{\bar{M}}_{n}$ *		2.0	2.0	2.2	2.1	2.3	2.1
Density	Kg/dm^3^	0.9066	0.9028	0.8978	0.8930	0.8903	0.8817
*T*g **	°C	−11	−12	−21	−22	−22	−27

* Data from Gel Permeation Chromatography (GPC) (Figures S2–S5); ** Data from Dynamic Mechanical Thermal Analysis (DMTA)

**Table 3:** Melting temperatures (TmI’, TmI, TmII), melting enthalpy (ΔHI), degree of crystallinity (C_DSC_, C_WAXD_) and half time of the II→I phase transformation (t ½) of the examined samples.

		PB0	PB1	PB2.4	PB2.9	PB3.8	PB6.8
TmII	°C	101.3	87.3	53.5	-	-	-
TmI	°C	117.3	104.6	87.1	73.9	64.1	48.9
TmI’	°C	-	-	37.2	36.1	38.8	38.3
ΔHI	*J/g*	72.3	63.3	47.7	46.7	40.0	25.5
C_DSC_	%	58	51	37	36	31	20
C_WAXD_	%	52	45	33	31	23	17
t ½	h	27.4	11.3	8.0	-	-	-

**Table 4:** Morphological parameters of the lamellar stacks obtained by SAXS analysis of the samples: long period (L), number of lamellae (N), thickness of the crystalline (Y), and amorphous layer (Z), along with their relative distributions, (σY/Y = σZ/Z, σL/L) and the degree of crystallinity by volume (Φ_SAXS_).

Sample	N	Y_1_	Z_1_	L_1_	Y_2_	Z_2_	L_2_	Φ_SAXS_
		*(nm)*	*(nm)*	*(nm)*	*(nm)*	*(nm)*	*(nm)*	*(%)*
PB0	∞	7.6	6.0	13.6	-	-	-	56
PB1	∞	5.3	7.3	12.6	-	-	-	42
PB2.4	∞	3.2	6.5	9.7	2.6	5.5	8.1	33
PB2.9	∞	2.8	7.1	9.9	2.2	5.5	7.7	28
PB3.8	∞	2.9	7.6	10.5	2.5	6.4	8.9	28
PB6.8	∞	3.3	10.7	14.0	2.7	8.7	11.4	24
		σ_Y1_/Y_1_	σ_Z1_/Z_1_	σ_L1_/L_1_	σ_Y2_/Y_2_	σ_Z2_/Z_2_	σ_L1_/L_2_	
		0.50	0.50	0.35	-	-	-	
		0.45	0.45	0.33	-	-	-	
		0.35	0.35	0.26	0.36	0.36	0.27	
		0.34	0.34	0.27	0.40	0.40	0.30	
		0.34	0.34	0.27	0.33	0.33	0.26	
		0.40	0.40	0.32	0.41	0.41	0.41	

**Table 5:** Comparison of morphological parameters obtained by SAXS and TEM analysis

Sample *	*SAXS*				*TEM*			
	*Y*		*L*		$\bar{Y}$	*Y_min_*	*Y_max_*	$\bar{L}$
	*(nm)*		*(nm)*		*(nm)*	*(nm)*	*(nm)*	*(nm)*
PB0	7.6		13.6		9.1	7.4	12.0	13.2
PB1	5.3		12.6		5.6	4.7	6.6	12.4
PB2.9	2.8	2.2	9.9	7.7	4.3	3.4	5.7	10.1
PB3.8	2.9	2.5	10.5	8.9	3.5	2.5	3.9	9.0
PB6.8	3.3	2.7	14.0	11.4	4.3	3.1	5.3	13.5

* Sample PB2.4, which, after aging, has a behavior overlapping that of sample PB2.9, has not been analyzed by TEM.

## Supplementary Materials

The following are available online at https://www.mdpi.com/2073-4360/11/7/1133/s1, Scheme S1: General polymerization process, Figure S1: FTIR spectra of samples PB1, PB2.4, PB2.9, PB3.8, PB6.8, Figure S2: GPC curve of sample PB0, Figure S3: GPC curve of sample PB1, Figure S4: GPC curves of samples PB2.4, PB2.9, PB3.8, Figure S5: GPC curve of sample PB6.8, Figure S6: DTA thermograms of PB2.4 at different heating rate, Figure S7: DTA thermograms of PB2.9 at different heating rate, Figure S8: DTA thermograms of PB3.8 at different heating rate, Figure S9: DTA thermograms of PB6.8 at different heating rate, Figure S10: SAXS pattern (dotted line) and trace calculated by fitting procedure (solid line) of PB0 Figure, Figure S11: SAXS pattern (dotted line) and trace calculated by fitting procedure (solid line) of PB2.4, Figure S12: SAXS pattern (dotted line) and trace calculated by fitting procedure (solid line) of PB2.9, Figure S13: SAXS pattern (dotted line) and trace calculated by fitting procedure (solid line) of PB3.8, Figure S14: SAXS pattern (dotted line) and trace calculated by fitting procedure (solid line) of PB6.8, S15: TEM image of PB0 (a), TEM image of the selected area and FFT signal (b), Figure S16: TEM image of P2.90 (a), TEM image of the selected area and FFT signal (b), Figure S17: TEM image of P3.8 (a), TEM image of the selected area and FFT signal (b), Figure S18: TEM image of P6.8 (a), TEM image of the selected area and FFT signal (b).
