# Supplementary material for: Poly (1-butene-ran-ethylene) Monomodal Copolymers from Metallocene Catalysts: Structural and Morphological Differences with Increasing Ethylene Content
Source: Polymers (Basel). 2019 Jul 3;11(7):1133. doi: 10.3390/polym11071133 (PMC6680423; doi:10.3390/polym11071133)
Supplement: Supplementary file 1 [file polymers-11-01133-s001.pdf]

Supplementary Material

Poly (1-butene-ran-ethylene) monomodal copolymers from metallocene catalysts: structural and morphological differences with increasing ethylene content

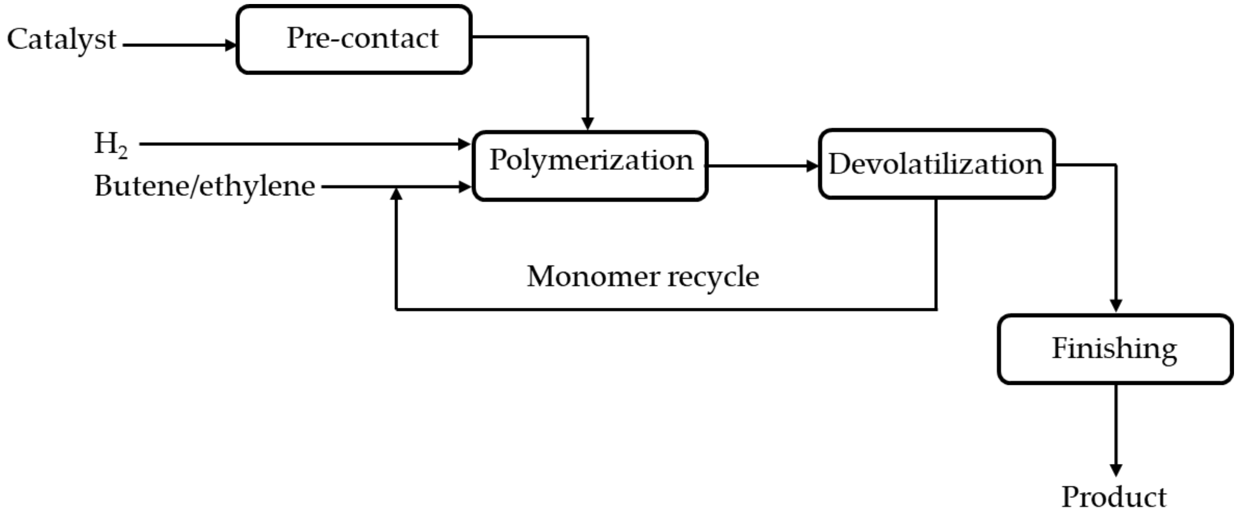

Scheme S1: General polymerization process

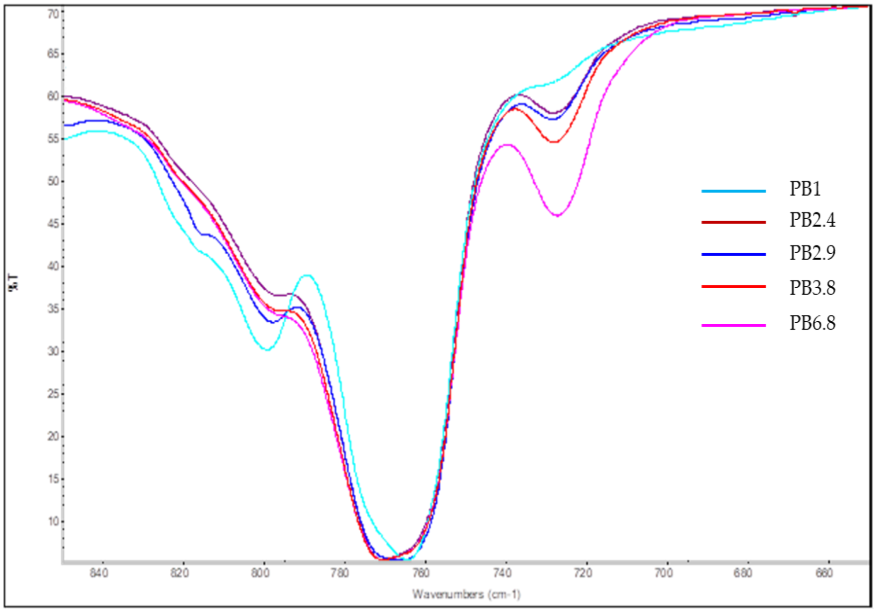

Figure S1: FTIR spectra of samples PB1, PB2.4, PB2.9, PB3.8, PB6.8 in the C2 region.

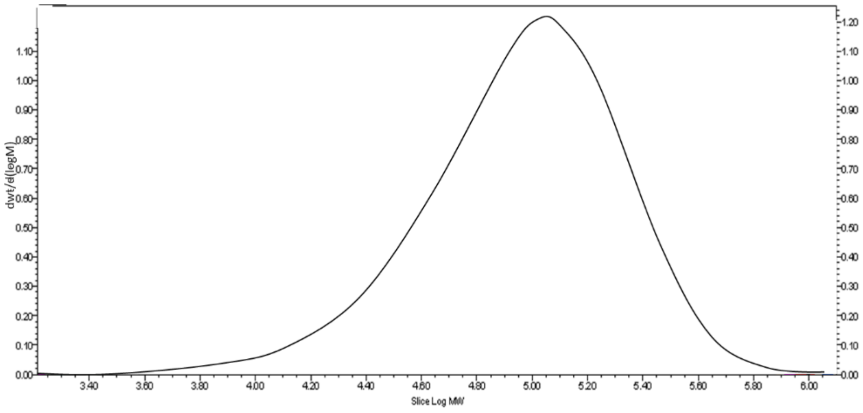

Figure S2: GPC curve of sample PB0.

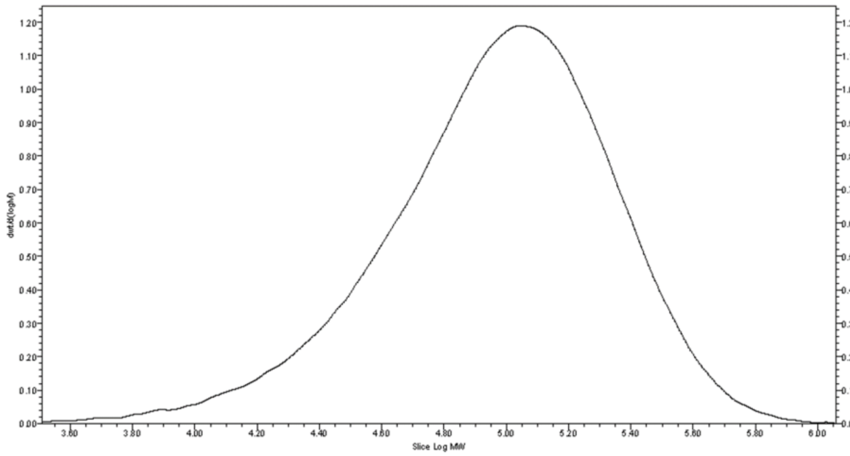

Figure S3: GPC curve of sample PB1.

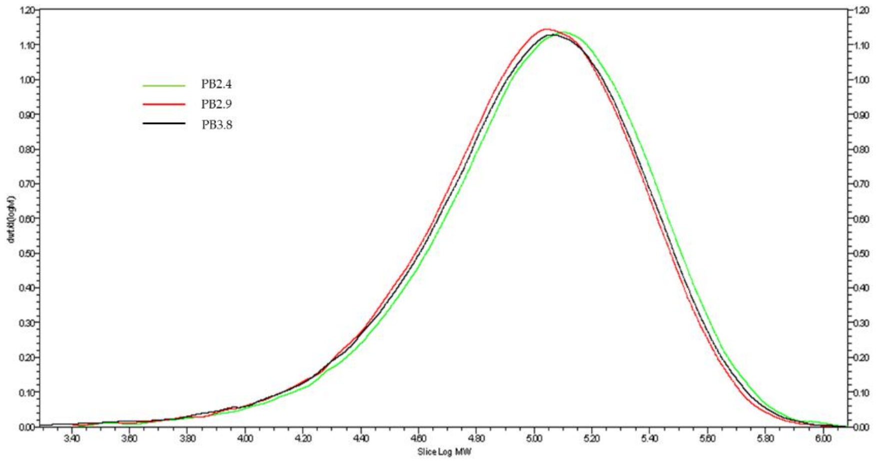

Figure S4: GPC curves of samples PB2.4, PB2.9, PB3.8.

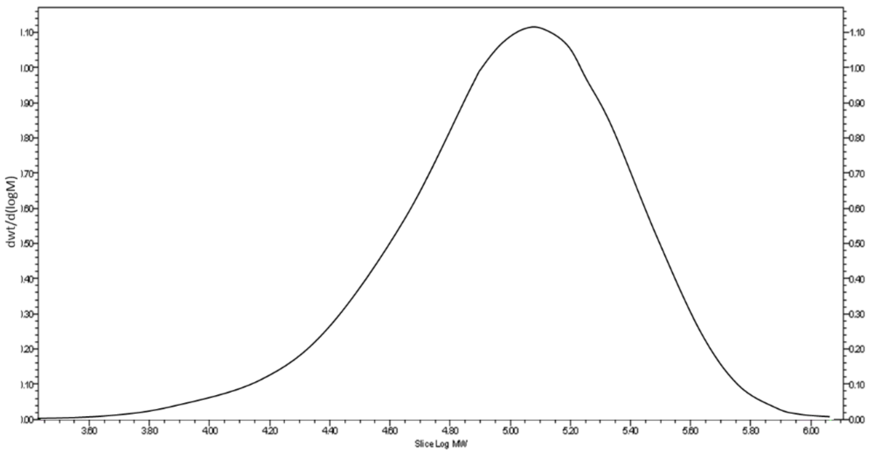

Figure S5: GPC curve of sample PB6.8.

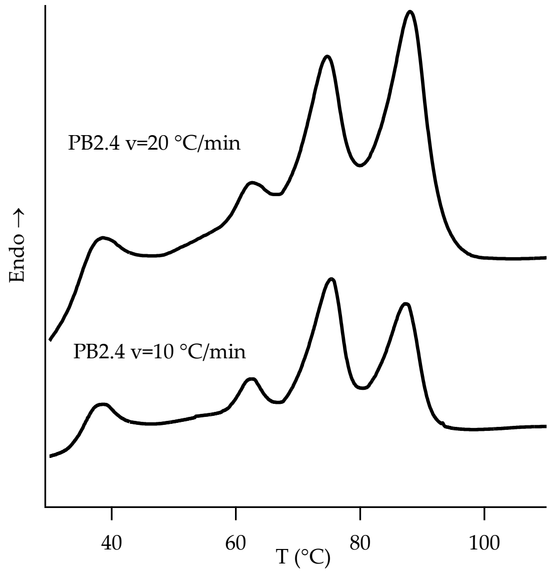

Figure S6: DTA thermograms of PB2.4 at different heating rate.

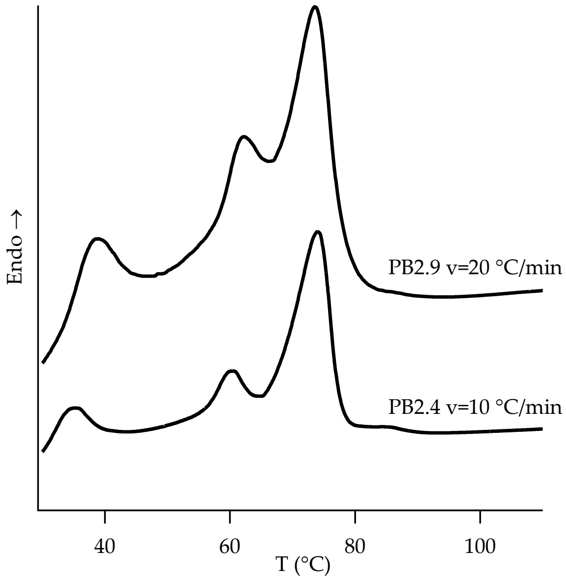

Figure S7: DTA thermograms of PB2.9 at different heating rate.

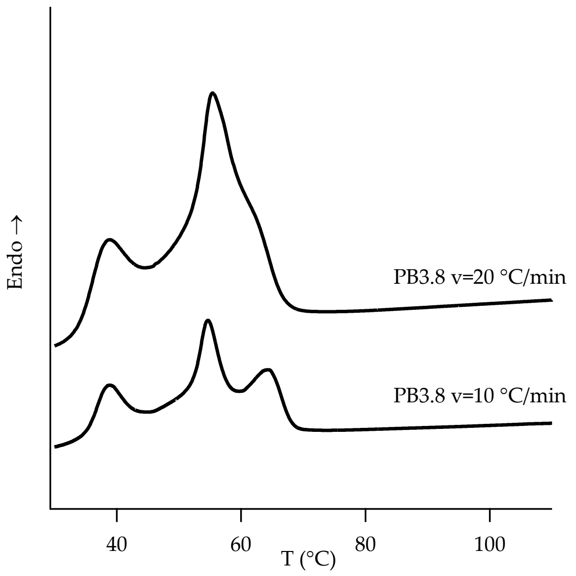

Figure S8: DTA thermograms of PB3.8 at different heating rate.

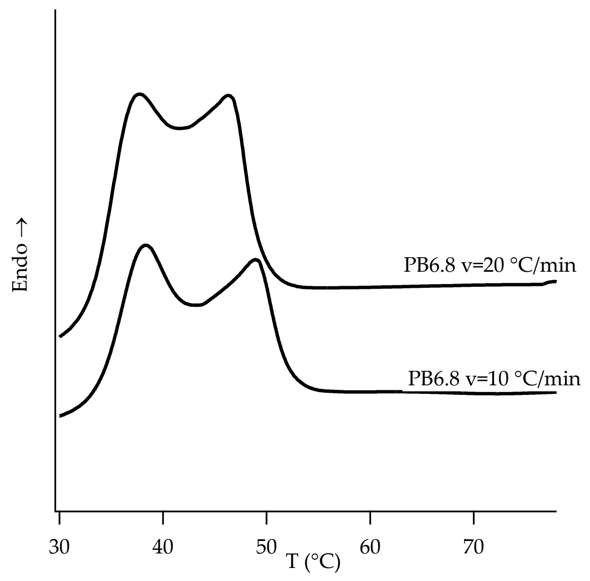

Figure S9: DTA thermograms of PB6.8 at different heating rate.

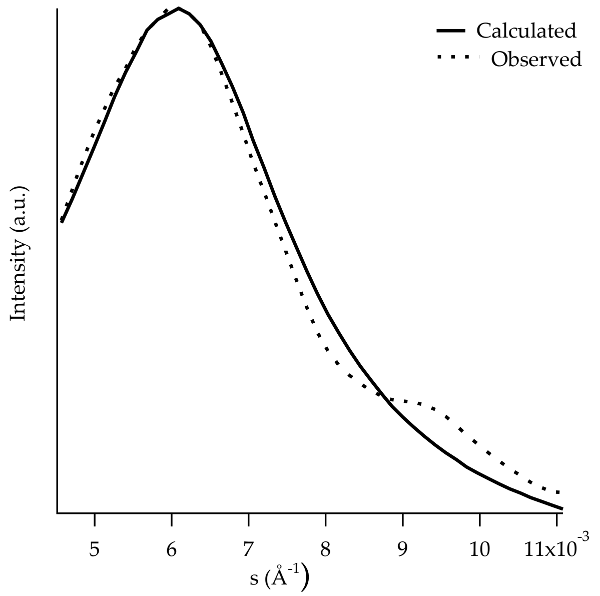

Figure S10. SAXS pattern (dotted line) and trace calculated by fitting procedure (solid line) of PB0

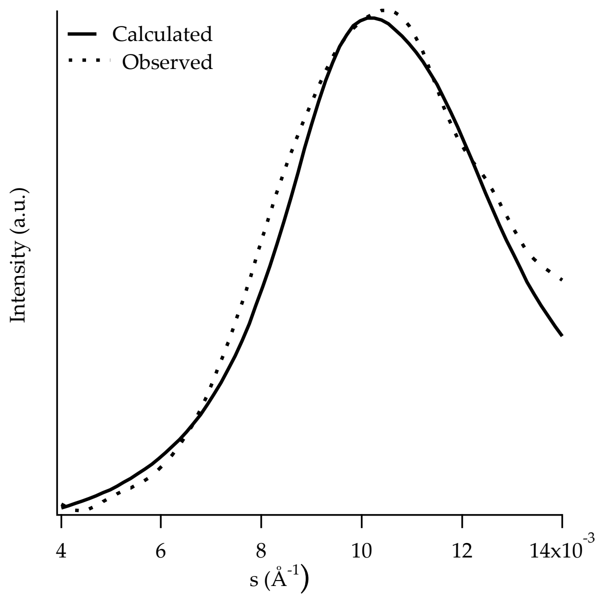

Figure S11. SAXS pattern (dotted line) and trace calculated by fitting procedure (solid line) of PB2.4

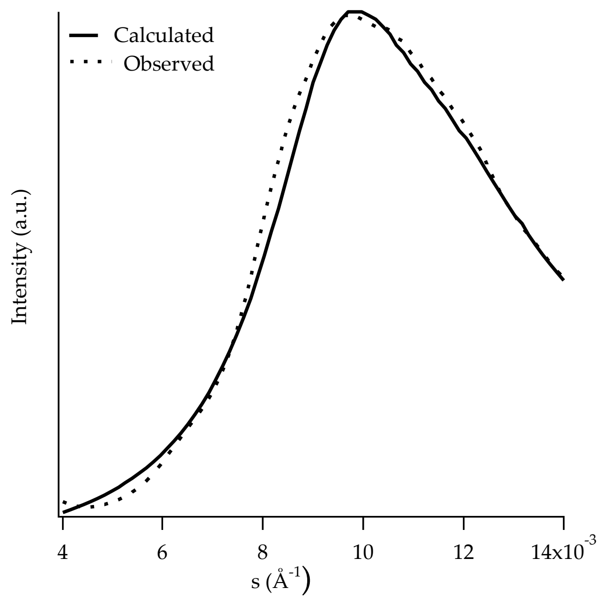

Figure S12. SAXS pattern (dotted line) and trace calculated by fitting procedure (solid line) of PB2.9

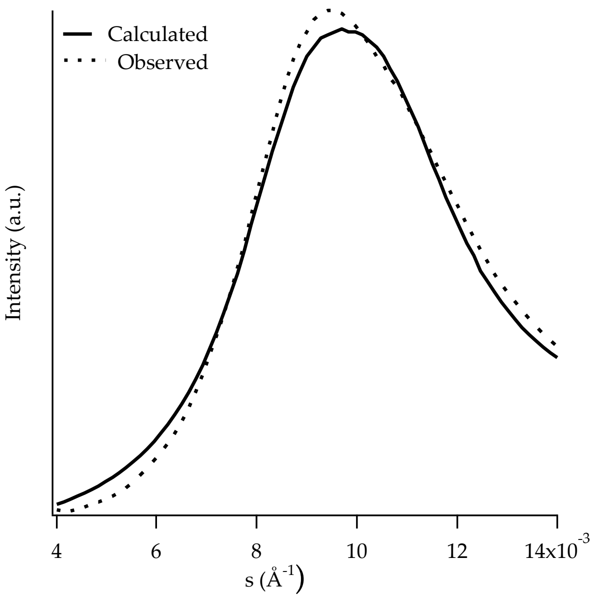

Figure S13. SAXS pattern (dotted line) and trace calculated by fitting procedure (solid line) of PB3.8

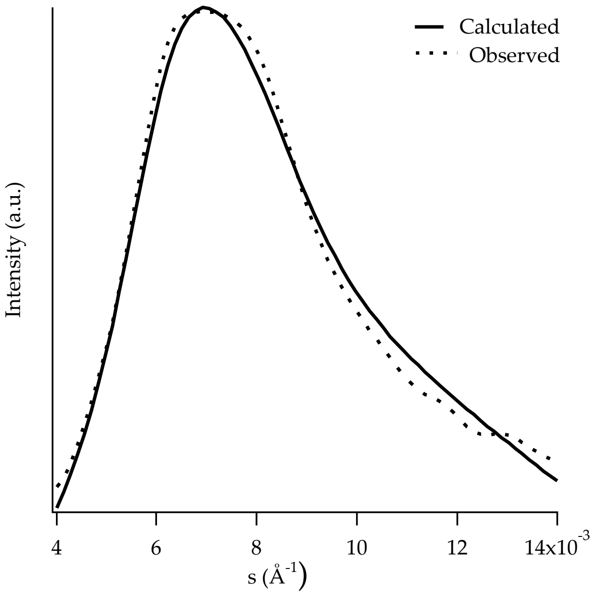

Figure S14. SAXS pattern (dotted line) and trace calculated by fitting procedure (solid line) of PB6.8

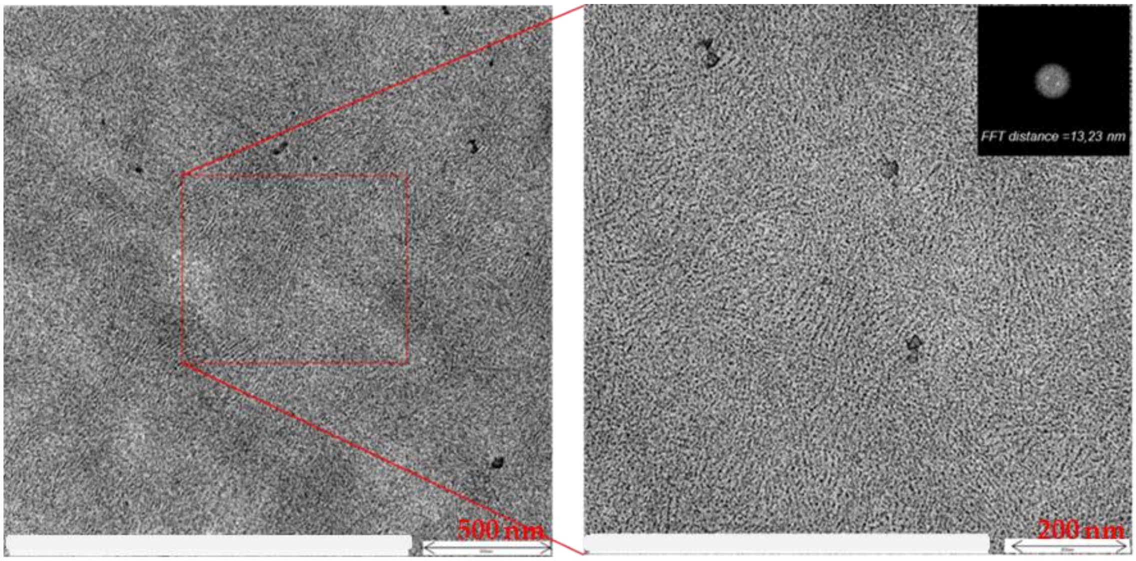

(a) (b)

Figure S15. TEM image of PB0 (a), TEM image of the selected area and FFT signal (b)

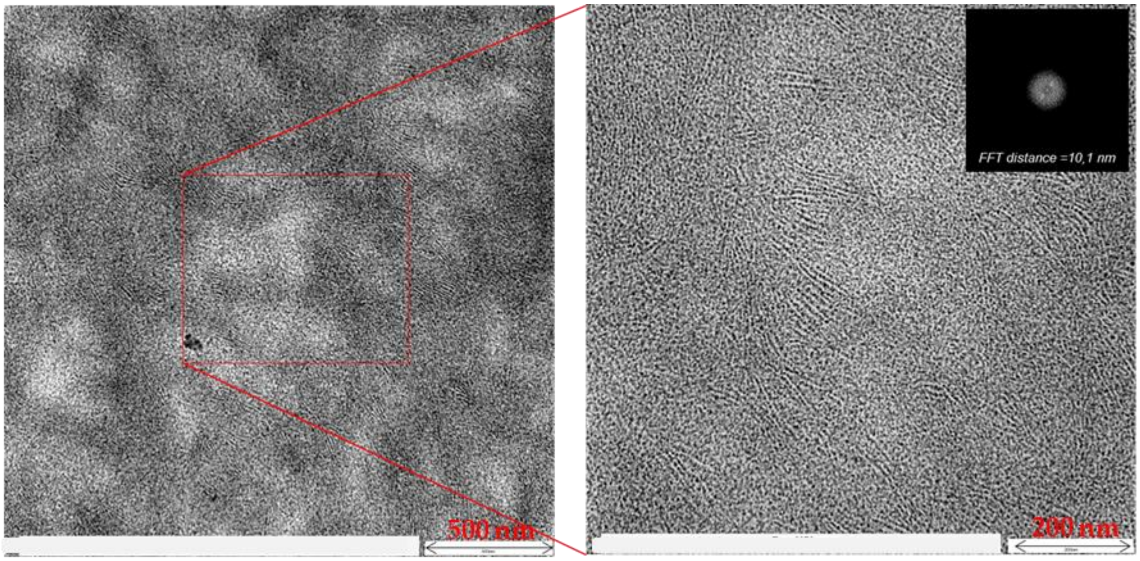

(a) (b)

Figure S16. TEM image of P2.9 (a), TEM image of the selected area and FFT signal (b)

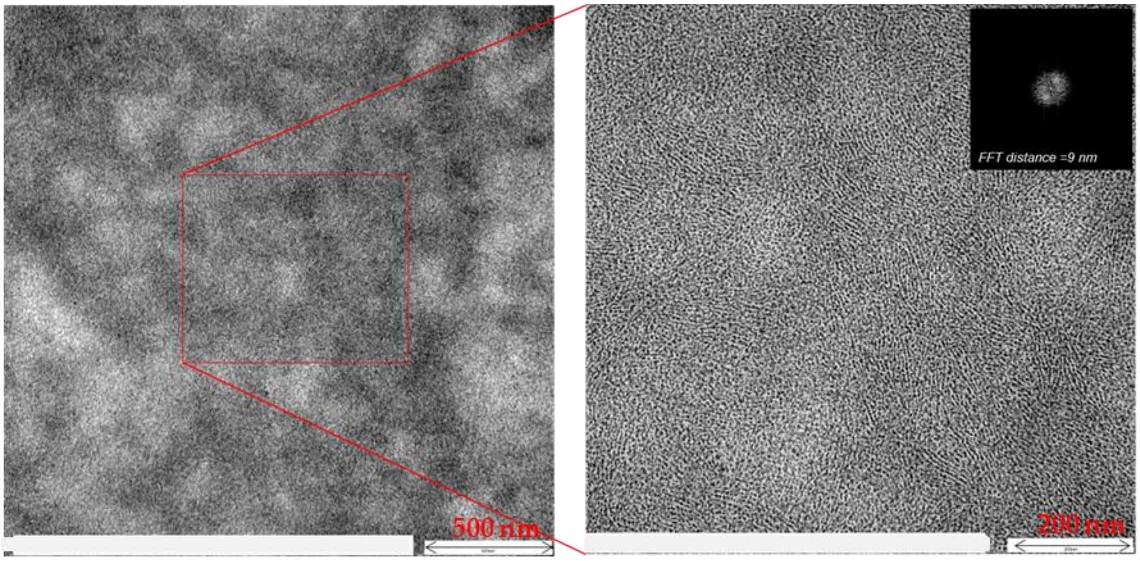

(a) (b)

Figure S17. TEM image of P3.8 (a), TEM image of the selected area and FFT signal (b)

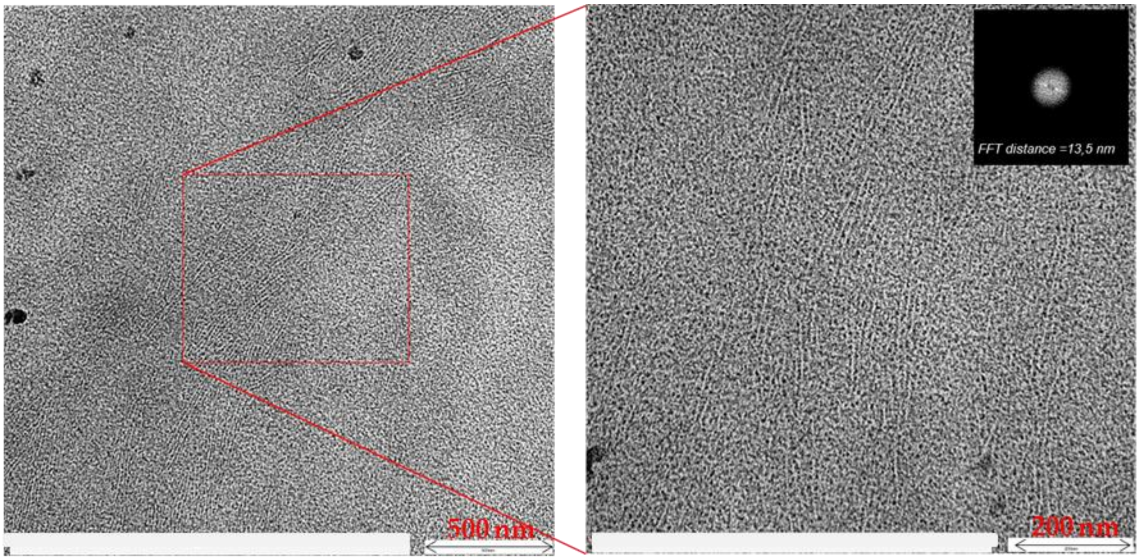

(a) (b)

Figure S18. TEM image of P6.8 (a), TEM image of the selected area and FFT signal (b)
